# The effect of landscape structure on dispersal distances of the Eurasian red squirrel

**DOI:** 10.1002/ece3.4806

**Published:** 2019-01-04

**Authors:** Suvi Hämäläinen, Karen Fey, Vesa Selonen

**Affiliations:** ^1^ Department of Biology, Section of Ecology University of Turku Turku Finland

**Keywords:** dispersal distances, Eurasian red squirrel, landscape ecology, movements, natal dispersal

## Abstract

Landscape structure can affect dispersal and gene flow in a species. In urban areas, buildings, roads, and small habitat patches make the landscape highly fragmented and can inhibit movement and affect dispersal behavior. Similarly, in rural forested areas, large open areas, such as fields, may act as barriers to movement. We studied how landscape structure affects natal dispersal distances of Eurasian red squirrels (*Sciurus vulgaris*) in an urban area and a rural area in Finland, by monitoring juvenile red squirrels with radio telemetry. We observed extremely long dispersal distances—up to 16 km—in the rural study area, but shorter distances—on average only half a kilometer—in the urban study area. The landscape structure affected the eventual dispersal paths; in the rural landscape, dispersers favored spruce dominated areas and avoided fields along their dispersal route, although they occasionally even crossed wide fields. In the urban landscape, squirrels preferred areas with deciduous or coniferous trees. The movement steps made by dispersers were longer in the more hostile landscape compared to forested areas. Despite these effects on movement path, the landscape structure only had a minor effect on straight line dispersal distances moved from the natal nest. In other words, individuals moved longer distances and were likely to circumvent barriers in their path, but this did not affect how far they settled from their natal home. This result indicates that, although landscape structure has obvious effects on movement, it still may have only a small effect on other aspects of the population, for example, gene flow.

## 1 INTRODUCTION

Landscape structure has a major influence on animal movement and distribution of species (Cote et al., 2017; Ims, 1995; Wiens, Stenseth, Van Horne, & Ims, 1993). In rural areas, forest management and agriculture are the main forces decreasing suitable habitat for forest species and isolating the remaining habitat patches. Both clear‐cutting and agricultural practices yield wide open areas that may inhibit movement of forest species (Bonte et al., 2012; Mader, 1984). Urban areas may be even more fragmented than rural areas; forest‐dwelling species are limited to inhabiting parks and other sites with fragmented tree areas, divided by roads and buildings. Like clear‐cuts and agricultural fields in rural areas, roads and buildings form barriers to movement in urban areas, inhibiting colonization of isolated habitat patches (Bonte et al., 2012; Lodé, 2000; Rondinini & Doncaster, 2002; Verbeylen, Bruyn, Adriaensen, & Matthysen, 2003).

Whether a species can move in a fragmented landscape and the populations remain viable depends on the gap‐crossing willingness of the dispersing individuals (Bakker & Van Vuren, 2004; Mäkeläinen, De Knegt, Ovaskainen, & Hanski, 2016; Selonen & Hanski, 2004). Dispersal refers to one‐way movement away from a home range. Mortality risk and other energetic costs related to gap crossing determine dispersal in an unsuitable habitat (Bonte et al., 2012; Fahrig, 2007). These dispersal costs differ in urban and rural areas; in the latter predation can be the main mortality risk, whereas in the former vehicle collisions may be important. However, in both urban and rural areas it has been noted that dispersing individuals may be more willing to cross gaps than non‐dispersing individuals (van Dyck & Baguette, 2005; Fey, Hämäläinen, & Selonen, 2016; Selonen & Hanski, 2006). For many mammals, the main dispersal period is when a juvenile abandons its natal home range (Clobert, Danchin, Dhondt, & Nichols, 2001; Wolff, 1994). Thus, natal dispersal is the main process behind gene flow and invasion potential of many species (Brommer, Wistbacka, & Selonen, 2017; Clobert et al., 2001; van Dyck & Baguette, 2005).

Arboreal mammals have very explicit habitat limitations and are thus interesting species to study regarding dispersal ability. They depend on forest habitats, which are often heavily fragmented in rural and, even more so, in urban areas. Many of these species may be reluctant to cross large forest gaps (Bakker & Van Vuren, 2004; van der Ree, Cesarini, Sunnucks, Moore, & Taylor, 2010) and may, therefore, be unable to colonize suitable and empty habitat patches (Bakker & Van Vuren, 2004; Delin & Andrén, 1999; Zollner, 2000). However, arboreal squirrels disperse long distances and cross unsuitable habitats, for example, roads, more often during natal dispersal than during movements within a home range (Fey et al., 2016; Selonen & Hanski, 2006; Verbeylen, Bruyn, & Matthysen, 2003). Only a few studies have examined the effect of landscape along dispersal route on movements of arboreal species (Merrick & Koprowski, 2017a; Selonen & Hanski, 2004; Verbeylen, Bruyn, Adriaensen, et al., 2003; Verbeylen, Bruyn, & Matthysen, 2003). Thus, we need better knowledge how landscape structure affects dispersal ability, and consequently, gene flow and invasion potential of arboreal species.

Here, we focus on the natal dispersal movements of the Eurasian red squirrel (*Sciurus vulgaris*) in fragmented environments. We conducted our study in two distinct landscapes, a rural area and an urban area of a city of 180,000 inhabitants. Our goal was to understand how an arboreal rodent moves through variable landscapes. We set out to determine (I) what are the dispersal distances of juvenile red squirrels, (II a) if juvenile squirrels favor a specific landscape on their dispersal routes (movement path taken during dispersal) and (b) if landscape structure affects step lengths (observed moves made within the dispersal route), and (III) how landscape structure affects the final dispersal distance of individuals (the straight line distance between natal and settlement sites). We hypothesize that dispersal distances vary in different landscapes due to the variation in resistance to species movement and predator risk of the landscapes. As red squirrels are a mainly arboreal species, we hypothesize that open areas along dispersal routes restrict dispersal distances of juvenile individuals. We also anticipate squirrels to favor moving in forested areas during their dispersal and the movement steps to be longer in the open areas than in the forested areas.

## 2 MATERIALS AND METHODS

### 2.1 Study area and data gathering

We collared and monitored in total 59 juvenile squirrels during 2012–2015, of which 32 were in the urban area of Turku, in southern Finland (60°27′05″N, 022°16′00″E), and 27 in the rural area of Southern Ostrobothnia, Finland (Kauhava, Lapua, and Lappajärvi region, hereafter referred to “Kauhava area”). The study areas were of approximately 6 km^2^ for urban Turku area and 900 km^2^ for rural Kauhava area. We collared squirrels at the approximate age of 1.5–2 months (178 ± 48 g in Turku, and 133 ± 44 g in the Kauhava area) with Biotrack radio collars weighing 5 or 8 g. Collars were fitted to tightness that allowed the neck of a juvenile individual to grow without being too loose to fall off. Squirrels were trapped from the ground with live traps, or by using a net to catch them from a nest box or cavity. After collaring, we immediately released squirrels at the place of their capture. Juvenile individuals were followed with a portable receiver (Biotrack sika) and 3‐element Yagi antenna approximately five times a week from early June to late September (resulting in an average of 63 ± 7 locations per individual in Turku area, and 33 ± 22 in Kauhava area). During active dispersal period, the squirrels were tracked more frequently. Squirrel locations were collected by determining either a single tree or a group of trees where the squirrel was located. In some occasions, when the landscape was difficult to enter (e.g., the squirrel located on a private yard), we used triangulation to determine the location of the animal. We continued the tracking in Turku study area throughout the following winter, with tracking intervals of one to two weeks, and in Kauhava area until the end of the year with 1 to 3 week intervals. Squirrel locations were made both in daytime and after dusk to gather both movement locations and nest locations.

### 2.2 Landscape data

In the Kauhava study area, the landscape was stratified into the following seven land use classes: young forest (including clear‐cuts), birch dominated forest, pine dominated forest, spruce dominated forest, built landscape (including buildings and roads), field, and water. Clear‐cuts were included in the young forest category, because they remain small in size in our study area, red squirrels occasionally move within clear‐cuts in the few trees left to the area (personal observation), and both areas have very restricted seed‐bearing ability. The land use classification was carried out with a land use map (on a 25 × 25 m pixel grid) based on the SLICE dataset, Landsat Images, and two forest classifications from 1997 and 2009. For a detailed description of land use map construction, see Morosinotto, Villers, Thomson, Varjonen, and Korpimäki (2017). In the Turku study area, a landscape map was made by manually digitizing different land use areas from an aerial orthophotograph (provided by: National Land Survey of Finland, 2008, ETRS‐TM35FIN, terrain resolution: 0.5 m) into a map at a scale of 1:800, with a minimum mapping unit of 1 m. We divided the landscape into six different land use classes: deciduous trees; coniferous trees; shrub or grass; waterway; building; and asphalt, gravel or sand (hereafter called “asphalt”). From these landscape maps, we calculated the area of the various land use classes within a 25 m buffer from the movement path for each individual for both study areas using ArcMap 10.1. We also calculated edge density by merging the original landscape type classes to contain only two different landscape types: wooded areas and other areas. Edge density represents the relationship between woodlot size and the amount of habitat edge.

### 2.3 Used and random movement paths

We analyzed whether squirrels avoid or favor certain landscape characteristics along their dispersal routes. In order to do this, we generated 100 random dispersal paths for each individual, by creating a special tool for ArcMap, to find out the available landscape through which the squirrels could have dispersed and compared that to the landscape of the original dispersal routes. We generated the random walk paths separately for each individual by using the original squirrel step lengths (distance between two consecutive observations) and randomizing the direction of movement for each step. Random paths started from the place of capture of the squirrel and ended at the distance of the squirrel's dispersal distance ±10%.

### 2.4 Landscape within dispersal routes

**Figure 1:**
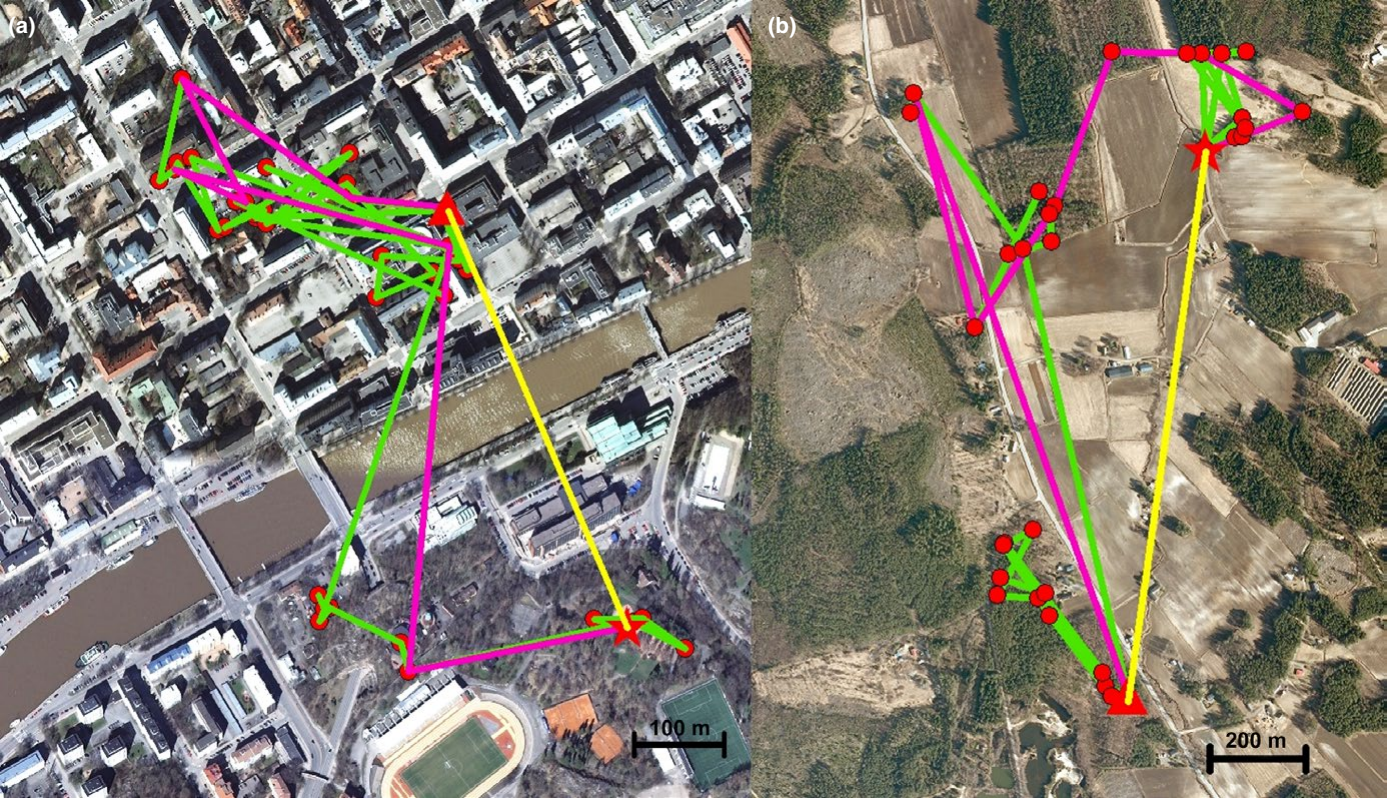
Examples of two individuals’ dispersal route (green) and the simplified dispersal route (purple), which includes only one location point from each visited area, in both the urban (a) and the rural (b) study area. Thus, simplified routes exclude the back‐and‐forth movement from one woodlot to another. Original squirrel locations are marked with red dots, the stars refer to the starting places of dispersal, and the triangles refer to the ending places of dispersal. The yellow lines between these locations are the straight line distances, that is, the dispersal distance

We measured the landscape composition along the dispersal route of juvenile squirrels in two ways: along the original dispersal route and along the simplified dispersal route. The *original dispersal route* consisted of all consecutive location points where the squirrel was observed during its dispersal period (an average of 63 ± 35 locations per individual in the urban area and 33 ± 22 in the rural area; the difference in number of locations reflects more intensive tracking in the urban study area due to logistic reasons; number of location fixes was not related to dispersal distances observed in this study). Thus, it included the back‐and‐forth movement the individual made while moving from one woodlot to another and back. The *simplified dispersal route* was created by excluding the back‐and‐forth movement by including only one location point from one visited woodlot. For this, we picked the first location where the squirrel was observed in each woodlot and formed a simplified dispersal route from these locations in order of visit (see Figure 1 for examples of dispersal routes). Next, we measured the amount of different landscape types and edge density around these two dispersal route types (25 m buffer around the movement path).

We then determined if the landscape along the original and simplified routes explain the *dispersal distance* (straight line distance between natal and settlement site) and the total *route length* of the simplified route (sum of step lengths of simplified route. An average of 7.9 ± 4.2 steps in the urban area and 3.3 ± 2.7 in the rural area, reflecting the higher tracking effort in the previous). The dispersal distance was recorded as a distance between the first observed nest of a juvenile individual and the last observed nest after its dispersal period. To calculate the dispersal distance, we only included the squirrels that survived until the end of the dispersal period (determined to be 15th of September) except in the case of three Kauhava squirrels, where the squirrels had already dispersed multiple kilometers from their natal site and were either killed or disappeared during the dispersal movements, leading to 18 individuals in Turku and 22 individuals in the Kauhava area. The total simplified route length was calculated as the total length of the simplified dispersal route, and it describes the total distance a squirrel has travelled when moving from one woodlot to another, including all the woodlots visited during dispersal (see Figure 1 for explanation of different dispersal routes and their lengths). We also studied the effect of landscape on the length of original singular squirrel steps observed, being the area between two consecutive location points. For this analysis, we omitted steps performed inside individuals’ natal or settlement site in order to focus only on dispersal movements, resulting in an average of 33.2 ± 25.7 steps per individual in the urban area and 11.7 ± 10.5 in the rural area. Here, we also used a buffer of 25 m to calculate landscape characteristics of each step.

### 2.5 Analyses

We tested if the dispersal distance, original dispersal route length, or route length of the simplified dispersal routes differed between the two study areas, or between sexes, by performing an analysis of variance, with the natural logarithm of dispersal distance as a response variable and the classes *area* and *sex* and their interaction as explanatory variables.

In order to understand habitat choices of red squirrels during dispersal, we performed a logistic regression to test whether the land use along dispersal routes differed from that of random routes (binomial distribution, GLIMMIX, SAS 6.1; separately for Kauhava and Turku study areas). The response variable was *used* (squirrel route) versus *available* (random routes), while the proportions of different land use categories were explanatory variables (i.e., the proportion of a specific land use class from the whole buffer around the route). *Squirrel individual* was set as a repeated factor, using generalized estimating equations, that is, we compared the landscape within route of a squirrel individual with 100 random routes created for that individual. We made separate models for both Kauhava and Turku study areas due to differences in landscape maps. In Kauhava, we made a separate model for the land use category *field*, to avoid strong collinearities between explanatory variables. In another model, the explanatory variables were *young forest*, *pine dominated forest*, *spruce dominated forest*, *built environment,* and *edge density*. In the Turku analyses, we included *asphalt* in a separate model, due to collinearities. In another model, we included *buildings*, *deciduous*, *coniferous,* and *edge density*. The above models were constructed so that the variance inflation factor would not be greater than 4 for any variable.

To study if the landscape structure resists movement steps taken by dispersing individuals, we performed, separately for Kauhava and Turku study areas, a generalized linear mixed model (GLIMMIX, SAS 6.1) with the squirrel step length as the response variable (Gaussian distribution). The explanatory variables included the landscape characteristics within a 25 m buffer around the step line. In addition, the *dispersal distance* and *duration of the step* (time between two tracking locations) were explanatory variables and *individual* was a random factor. We tested whether the width of the buffer around the step influences on the results of Turku study area, where the steps are relatively short, by conducting the analysis using a 10 m buffer around steps. The results did not differ from the results of the 25 m buffer (analysis not shown).

Finally, we tested whether the landscape along the dispersal route (original or simplified route) affects dispersal distances or route length, to study if the dispersal distances are restricted by the landscape structure. We created linear mixed models with *dispersal distance* or *route length* as a response variable and landscape parameters being explanatory variables (GLIMMIX, SAS 6.1). For this analysis, we combined the data of both of our study areas, because of the low number of individuals. *Study area* was included as a class variable in the models. We also grouped the landscape classes of both study areas in order to have the following similar landscape variables in both study areas: *preferred habitat*, *unpreferred habitat*, *open*, *water,* and *edge density*. We conducted separate models for open areas in each model to achieve VIF‐values under 4.

## 3 RESULTS

### 3.1 Dispersal distances

**Figure 2:**
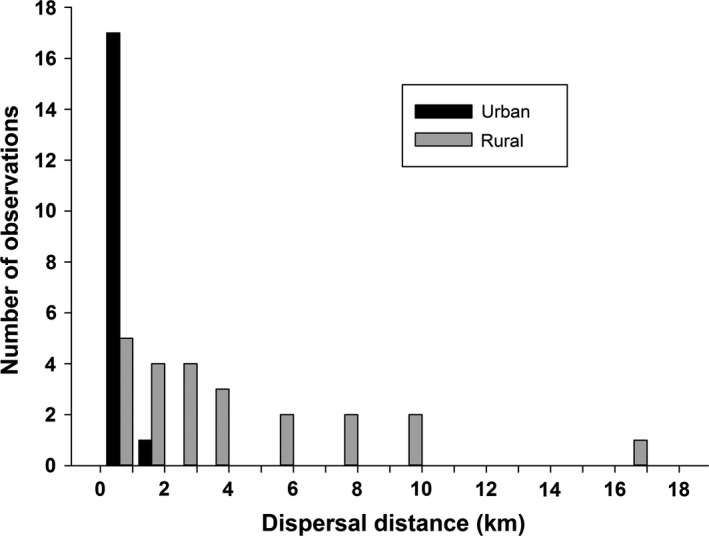
The distribution of dispersal distances in the rural (gray bars) and urban (black bars) study areas (in km)

The dispersal distances were significantly longer in the rural Kauhava region than in the urban Turku area (*n* = 40; Table 1, Figure 2). Also the original routes juvenile squirrels travelled during dispersal were longer in rural study area, but this was not observed for simplified routes (see section 2 for definition of route; Table 1). Dispersal distances did not differ between sexes (*F*_2,36_ = 1.76, *p* = 0.19), and there was no significant interaction between sex and study area (*F*_1,2_ = 0.52, *p* = 0.47) for dispersal distance. However, female juvenile squirrels had slightly longer simplified route lengths than males (*F*_1,36_ = 5.46, *p* = 0.02; Table 1). There was no significant interaction between sex and study area for either original (*F*_1,26_ = 1.60, *p* = 0.22) or simplified (*F*_1,35_ = 1.76, *p* = 0.19) route length.

**Table 1:** Dispersal distance, original route length, and simplified route length (in meters) for male and female red squirrels in urban Turku and rural Kauhava study areas in Finland. Results of analysis of variance

	Turku		Kauhava		Test between study areas	
	Male	Female	Male	Female	F	p
Dispersal distance						
Average ± SD	427 ± 415	440 ± 263	2469 ± 2352	4733 ± 4486		
Sexes combined	431 ± 363		3638 ± 3774		37.07	**< 0.001**
Original route						
Average ± SD	6744 ± 3253	6079,8 ± 1923	8526 ± 3905	10047 ± 6817		
Sexes combined	6459 ± 2731		9822 ± 5321		4.81	**0.04**
Simplified route						
Average ± SD	1827 ± 1239	6079,8 ± 1923	2355 ± 2335	5662 ± 5891		
Sexes combined	1945 ± 1268		4412 ± 4853		2.54	0.12

Significant *p*–values are highlighted with bold.

### 3.2 Landscape composition along dispersal route compared to random routes

In the Kauhava region, squirrels’ dispersal routes (*n* = 27) included more young forest and spruce dominated forest and less field than the random routes (Table 2). Squirrel routes also contained more landscape with high edge density than the random routes (Table 2). In the Turku area, squirrel dispersal routes (*n* = 26) had more buildings and coniferous and deciduous trees compared to the random routes (Table 2).

**Table 2:** The logistic regression analysis of the landscape composition (% for asphalt, building, deciduous and coniferous; meters per hectare for edge density) within dispersal routes (25 m buffer) of juvenile squirrels compared to random routes available in the landscape. Estimate presented as log odd values, negative parameter estimate indicates avoidance. There were 26 dispersal routes in urban Turku and 27 in rural Kauhava. The number of random routes was 100 for each individual (see section 2)

	Used route Average ± *SD*	Random route Average ± *SD*	Estimate	*SE*	*z*	*p*
Turku						
Asphalt	32 ± 9	37 ± 8	−0.07	0.05	−1.65	0.10
Building	12 ± 7	15 ± 7	0.18	0.06	3.06	**0.002**
Deciduous	35 ± 12	28 ± 10	0.12	0.04	3.39	**0.0007**
Coniferous	4 ± 13	2 ± 6	0.11	0.04	2.75	**0.006**
Edge density	670 ± 126	592 ± 120	0.005	0.003	1.94	0.053
Kauhava						
Young	35 ± 13	25 ± 14	0.06	0.02	2.71	**0.007**
Pine	25 ± 13	17 ± 13	0.04	0.02	1.67	0.09
Spruce	7 ± 5	5 ± 4	0.11	0.05	2.27	**0.02**
Field	24 ± 17	44 ± 24	−0.04	0.01	−3.73	**0.0002**
Built	8 ± 7	7 ± 5	0.06	0.05	1.1	0.27
Edge density	69 ± 44	48 ± 26	0.03	0.008	3.46	**0.0005**

Significant *p*‐values are highlighted with bold.

### 3.3 Landscape effects on step length

In the rural Kauhava region, we found that the observed steps of squirrels (*n* = 332) were shorter when there was more pine and spruce dominated forests along the squirrel step, whereas the amount of field had a positive effect on step length; the more field space between the two consecutive squirrel locations, the longer the distance between these points was (Table 3). The duration of the step (time between two tracking locations; time in Table 3) and dispersal distance had a positive effect on the step length in Kauhava (Table 3). In the urban Turku study area (*n* = 709), more deciduous trees along the squirrel steps led to shorter steps, whereas more asphalt led to longer steps. In Turku we did not find any effect of dispersal distance or time on the length of squirrel steps (Table 3), perhaps due to more intensive tracking in Turku than in Kauhava.

**Table 3:** The generalized linear mixed model analysis of the effect of landscape composition on movement step length during dispersal of juvenile red squirrels. Negative parameter estimate indicates shorter steps. Landscape variables measured within a 25 m buffer around steps

	Estimate	*SE*	*z*	*p*
Turku				
Asphalt	0.02	0.003	5.33	**<0.0001**
Building	−0.0005	0.006	−0.08	0.94
Deciduous	−0.02	0.004	−4.64	**<0.0001**
Coniferous	−0.03	0.02	−1.67	0.10
Grass	−0.002	0.005	−0.34	0.73
Dispersal distance	3.29E−6	0.00004	0.01	0.99
Time	0.02	0.02	0.92	0.36
Kauhava				
Pine	−0.01	0.003	−3.01	**0.003**
Spruce	−0.01	0.005	−3.21	**0.002**
Field	0.01	0.004	3.51	**0.005**
Built	−0.01	0.006	−1.43	0.15
Dispersal distance	0.00006	0.00001	5.25	**<0.0001**
Time	0.04	0.008	5.55	**<0.0001**

Significant *p*‐values are highlighted with bold.

### 3.4 Landscape effects on dispersal distance and route length

Contrary to that observed for step lengths, the habitat variables did not affect dispersal distance (straight line dispersal from natal to settlement) or route length, with the exception of edge density (Table 4). The more edge of forested habitat there was along both original route and simplified route, the shorter the dispersal distances were (Table 4).

**Table 4:** The generalized linear mixed model analysis of the effect of landscape composition to dispersal distance (straight line distance between natal and settlement sites) and simplified route length (path taken during dispersal, excluding the back‐and‐forth movement). Negative parameter estimate indicates shorter distance. Landscape composition measured both along original route and simplified route. The latter excludes the movement back and forth between different forest sites. Study areas were combined for this analysis, see section 2

	Estimate	*SE*	*z*	*p*
Dispersal distance: Landscape variables measured along original route				
Preferred	−0.02	0.12	−1.39	0.17
Unpreferred	−0.001	0.02	−0.08	0.94
Open	0.005	0.01	0.44	0.66
Edge density	−0.005	0.002	−2.63	**0.01**
Dispersal distance: Landscape variables measured along simplified route				
Preferred	−0.009	0.01	−1.00	0.33
Unpreferred	−0.05	0.03	−0.48	0.63
Open	0.004	0.01	0.41	0.68
Edge density	−0.005	0.002	−2.46	**0.02**
Simplified route length: Landscape variables measured along simplified route				
Preferred	−0.007	0.008	−0.88	0.39
Unpreferred	0.02	0.03	0.77	0.45
Open	0.005	0.008	0.64	0.52
Edge density	−0.003	0.001	−1.87	0.07

Significant *p*‐values are highlighted with bold.

## 4 DISCUSSION

In this study, we observed long dispersal distances for red squirrels in the rural environment, but shorter in the urban environment. The landscape structure affected dispersing red squirrels’ dispersal paths; individuals avoided moving through and took longer steps in the unfavorable environment. Despite these effects on movement path, the final straight line dispersal distance remained mostly unaffected by the landscape structure.

Observed straight line dispersal distances of juvenile red squirrels were extremely long in the rural Kauhava study area (mean distance: 3.6 km, max distance: 16 km), especially when considering that some of the long‐dispersed study individuals disappeared during their dispersal movements, meaning that the actual dispersal distances would have been even longer than the observed ones. Previous studies have shown approximately 250–1,000 m dispersal distances for rural red squirrels in Belgium, depending on forest type (Wauters & Dhondt, 1993; Wauters, Verbeylen, Preatoni, Martinoli, & Matthysen, 2010). Similarly to Wauters, Preatoni, Martinoli, Verbeylen, and Matthysen (2011), we did not observe sex bias in red squirrel dispersal distances. However, simplified route lengths were longer for females than for males, which indicate there may be some sex bias in dispersal behavior of red squirrels. Indeed, for some arboreal squirrels there is also observed sex‐biased dispersal patterns (Koprowski, 1996; Merrick & Koprowski, 2017a; Selonen, Hanski, & Painter, 2010). For example, Merrick and Koprowski (2017a) have found that the body mass of the mother affects differently to male and female juvenile dispersal distances in Mt. Graham red squirrel.

The dispersal distances clearly differed between our study sites, being 8–9 times longer in the rural area compared to those in the urban study area. In our study, the dispersal distances of the urban study area (mean: 431 m) correspond to the ones observed in Belgian rural areas (Wauters & Dhondt, 1993; Wauters et al., 2010). The cause of the extensive difference in dispersal distances between our urban and rural squirrels remains uncertain, but in support for our results urban chipmunks are observed to reduce locomotion compared to their rural counterparts (Lyons, Mastromonaco, Edwards, & Schulte‐Hostedde, 2017). If the squirrels in our study would have been restricted to disperse in urban landscape, the analysis of landscape structure effect on dispersal distance or the research conducted by Fey et al. (2016) should have shown some evidence of it (see discussion below, see also Selonen, Fey, & Hämäläinen, 2018). Instead, a potential reason for the observed differences between urban and rural area is related to differences in settlement decisions. Resource availability and density of individuals determine settlement decisions of dispersing squirrels (Merrick & Koprowski, 2017a; Selonen & Hanski, 2012) and are known to be potentially higher in the urban area than in rural areas for red squirrels (Haigh, Butler, O'Riordan, & Palme, 2017; Jokimäki, Selonen, Lehikoinen, & Kaisanlahti‐Jokimäki, 2017). In the urban area, food availability is more stable than in rural areas, as there are multiple tree species and additional feeding provided by humans. In the rural area, squirrels are extremely dependent on coniferous seed crops that can vary heavily between years (Jokimäki et al., 2017). When resources are scattered, home ranges of adults are large and thus the dispersal distances for juvenile squirrels may need to be long in order to leave the mother's home range. On the other hand, when individual home ranges are scattered in the landscape, dispersers need to move long distances to locate potential mates. Unfortunately, we did not have data on squirrel densities or site occupancies within the dispersed landscapes, but earlier studies indicate that occupancy status of forest sites is important for settlement decisions of arboreal squirrels (Boutin, Tooze, & Price, 1993; Lurz, Garson, & Wauters, 1997; Wauters et al., 2010) and also canopy cover and forest structure affect habitat selection (Merrick & Koprowski, 2017b; Selonen & Hanski, 2012).

In the analysis comparing used and available dispersal routes, edges were preferred by juvenile squirrels. Perhaps this result reflects individuals spending time and/or moving along edges (see e.g., Latham, Latham, Boyce, & Boutin, 2011). Indeed, earlier studies have observed that red squirrels may prefer edge habitats (Dylewski, Przyborowski, & Myczko, 2016; Turkia, Korpimäki, Villers, & Selonen, 2018). In the case of the red squirrel, the reason to spend time at forest edges may be due to the possibly larger cone production of spruces growing on edges and getting more light for their growth (Dylewski et al., 2016). The lack of preference for coniferous trees along squirrel steps in our urban study area may be due to the very small proportion of coniferous trees in city of Turku. In studies of habitat use of squirrels in urban landscapes, it has been observed that squirrels favor green, forested areas (Bonnington, Gaston, & Evans, 2014; Hämäläinen, Fey, & Selonen, 2018). In our study, urban red squirrels moved in closer proximity to buildings than it would be expected at random, although when considering mean values of squirrel routes and random routes, without taking into account squirrel individuals, the result was the opposite (Table 2). In addition, when the variable *buildings* were treated alone it had negative effect, but when together with other variables it turned positive. This likely reflects that modeling with several covariates allows to setting apart confounding effects. That buildings were actually preferred is supported by our previous analysis, where the habitat use of squirrels in an urban area was studied (Hämäläinen et al., 2018). It is possible that squirrels moved through built areas in order to utilize supplement feeding provided by humans (Jokimäki et al., 2017). In the rural area, the observed preference for young forests was surprising. These stands have limited cone production, but study squirrels sometimes spent time in pine sapling stands in the Kauhava region, and some had also nests in these stands.

Squirrels moved, as expected, longer movement steps to find a suitable habitat patch when there was unfavorable habitat along the route. This might increase mortality of dispersers, but in our data, mortality occurrences during dispersal are rare (Fey et al., 2016). In the Turku area, squirrel steps were shorter within deciduous habitat, squirrels likely spending more time in that habitat, whereas, we did not find such preference for coniferous forest. Edge density had a negative effect on dispersal distance, which means that squirrels moved shorter distances when there were more edges present. As there are dominance hierarchy observed in red squirrel with juvenile individuals ranking lower than adults (Wauters & Dhondt, 1992; Wauters, Gurnell, Preatoni, & Tosi, 2001), it is possible that interior habitats are already occupied by resident adult individuals expelling juveniles away from these areas, leading juveniles to use more edge habitat. The negative relationship between edge density and dispersal distance could also result from fragmentation of habitats preventing movement of juvenile individuals. However, this hypothesis seems unlikely in our case (see also Fey et al., 2016) because the amount of unpreferred habitat along the dispersal routes did not affect dispersal distances. We think the association with edges observed in this study simply reflects the preference for edge habitats in our study areas (Turkia et al., 2018).

Despite the observed effects on step lengths, the straight line dispersal distance moved from natal site was not clearly affected by the landscape structure (except for the effect of edges). For example, even though the squirrels avoided fields in the rural landscape, the amount of open areas did not affect dispersal distances in this region. It has been previously studied in other squirrel species that the gap size or crossing distance does not affect crossing behavior in open areas for adult individuals (Bakker & Van Vuren, 2004; Bowman & Fahrig, 2002). For example, the American red squirrel (*Tamiasciurus hudsonicus*) avoids open areas created by clear‐cuts, but the probability to cross a gap increases when the circuitous route distance along the forest increases (Bakker & Van Vuren, 2004). Our results support this, as the amount of field on the dispersal route of juvenile squirrels did not affect dispersal distance; in other words, squirrels likely circumvented some agricultural areas. However, even the large fields can be crossed by dispersing red squirrels (Verbeylen, Bruyn, & Matthysen, 2003). In our study, the largest agricultural gap crossed by a juvenile individual was, at its narrowest, an approximately 3 km wide open field, with a river running in the middle of the area and no circuitous route available. This highlights the great movement potential of red squirrel juveniles.

Thus, the long dispersal distances of red squirrels seem to be driven by other forces than landscape structure preventing or enhancing movement of individuals. Similarly, Wauters et al. (2010) concluded that habitat fragmentation does not affect dispersal distances of red squirrels. Possible candidates driving long‐distance dispersal in red squirrels include the resource situation and population density (Lurz et al., 1997; Wauters & Dhondt, 1993). Irrespective of the causes behind dispersal distance our study may guide conservation managers of arboreal squirrels. Knowledge of dispersal ability of other arboreal squirrel species is deficient, but there are indications of great movement potential of invasive squirrel species (see Selonen & Mäkeläinen, 2017 for a review), like gray squirrel (*Sciurus carolensis*) and *Callosciurus* species. Also, Siberian flying squirrels are known to be effective dispersers (Selonen & Hanski, 2004, 2012). The findings of our study about the limited effect of landscape structure on squirrel dispersal can help on estimating the movement ability and spread of invasive squirrel species and, thus, benefit conservation planning of the native squirrel species. The knowledge of juvenile movements in relation to landscape structure provides us tools to estimate species distribution and spread in a changing environment.

We conclude that landscape structure has an obvious effect on movement patterns of juvenile red squirrel individuals, but it may have only a limited effect on dispersal distance and thus, on gene flow and population dynamics of the species. Juvenile red squirrels are effective dispersers having potential for very long movements, and their dispersal is not significantly restricted by a fragmented environment. Still, dispersal behavior potentially varies substantially in different environments. This indicates that more knowledge on effect of, for example, variation in resource levels and density of individuals on movement decisions are needed to understand dispersal behavior of the species.

## CONFLICT OF INTEREST

None declared.

## AUTHOR CONTRIBUTION

VS conceived the ideas and designed methodology; SH, VS, and KF collected the data; SH and VS analyzed the data; SH led the writing of the manuscript. All authors contributed critically to the drafts and gave final approval for publication.

## Data Availability

Data available from the Dryad Digital Repository, https://doi.org/10.5061/dryad.b0c6j7k.
